# The effects of blood transfusion on renal functions in orthopaedic surgery

**DOI:** 10.12669/pjms.314.7884

**Published:** 2015

**Authors:** Ismail Safa Satoglu, Serkan Akcay, Levent Horoz, Erol Kaya, Ahmet Karakasli, Eyad Skiak, Onur Basci

**Affiliations:** 1 Ismail Safa Satoglu, Department of Orthopaedics & Traumatology, Dokuz Eylul University, Faculty of Medicine, Izmir, Turkey; 2 Serkan Akcay, Department of Orthopaedics & Traumatology, Kurtköy Ersoy Hospital, Istanbul, Turkey; 3 Levent Horoz, Department of Orthopaedics & Traumatology, Dokuz Eylul University, Faculty of Medicine, Izmir, Turkey; 4 Erol Kaya, Department of Orthopaedics & Traumatology, Dokuz Eylul University, Faculty of Medicine, Izmir, Turkey; 5 Ahmet Karakasli, Department of Orthopaedics & Traumatology, Dokuz Eylul University, Faculty of Medicine, Izmir, Turkey; 6 Eyad Skiak, Karatas Hospital, Izmir, Turkey; 7 Onur Basci, Department of Orthopaedics & Traumatology, Dokuz Eylul University, Faculty of Medicine, Izmir, Turkey

**Keywords:** Blood transfusion, Renal functions, Orthopaedic surgery

## Abstract

**Objective:**

The effects of perioperative blood transfusion on renal functions have been studied in various studies. In this study, we investigated the effects of blood transfusion on postoperative kidney functions in patients who underwent orthopaedic surgeries.

**Method:**

Total 136 patients who were operated for several orthopedic pathologies between June 2013 and December 2014 were evaluated. The patients were divided into two groups according to the amounts of blood transfusion. Ninety five patients (69.8%) who were transfused less than 3 units were included in Group 1 and 41 patients (30.2%) who received 3 and more units of blood were included in Group 2.

**Results:**

There were no statistical difference between the two groups in terms of preoperative gender, hypertension, diabetes mellitus, chronical renal failure and smoking habbits (P > 0.05). No statistical differences between the groups were seen in terms of postoperative hospital stay, pulmonary and other complications as well as mortality (P > 0.05). When the two groups were compared for blood parameters showing postoperative renal and other system functions, no statistical differences were detected (P > 0.05).

**Conclusion:**

Blood transfusion does not have negative effects on postoperative BUN and creatinine levels in patients operated for orthopaedic pathologies.

## INTRODUCTION

Approximately 10% of all perioperative blood transfusion in USA is due to surgeries performed for orthopaedic pathologies. Among these orthopaedic surgeries performed, joint replacement arthroplasty constitudes the majority with 39%.^1^,^2^ Increased rates of morbidity as well as mortality is reported due to increased perioperative blood transfusion in orthopaedic operations consistent with increased blood transfusion complications in other fields of surgery.^1^-^4^

Leading complications due to perioperative blood transfusion in patients undergoing surgical interventions are hemolytic and allergic reactions, transfusion-associated acute lung injury, transfusion-associated circulatory overload, graft-versus-host disease and infection.^1^,^3^-^8^ Another complication of perioperative blood transfusion is renal dysfunction as specified in several studies.^9^-^11^

However, our literature search did not yield many studies investigating the effects of perioperative blood transfusion on kidney functions in orthopaedic operations. In this study, we investigated how perioperative blood transfusion affected renal functions in patients who underwent operations due to orthopaedic pathologies.

## METHOD

Total 136 patients operated in Dokuz Eylul University Hospital, Department of Orthopaedics and Traumatology for various orthopaedic pathologies between June 2013 and December 2014 were retrospectively evaluated. Patient file records were utilized after the approval of the ethical committee. Age, gender, comorbidities like hypertension, diabetes mellitus, chronic obstructive lung disease, chronic renal disease and smoking habbits as well as types of operations, anesthesia, preoperative and postoperative blood glucose levels, perioperative amounts of blood transfusion and postoperative morbidities and mortalities were recorded. One hundred thirty six operated patients included 47 total hip arthroplasties, 35 bipolar hip arthroplasties, 18 total knee arthoplasties, 2 shoulder arthroplasties, 16 open reduction and internal fixations, 9 intramedullary nailings and 9 posterior spinal intrumentations.

Ninety five patients (69.8%) who were transfused less than 3 units perioperatively were classified as Group 1 and 41 patients (30.2%) who received 3 and more units of blood transfusion were classified as Group 2. Two groups were compared especially for postoperative renal functions. Patients were transfused when postoperative Hct levels were below 24%. Patients who experienced renal dysfunction were consulted to Nephrology Department and treated according to their order. None of the patients needed hemodialysis. Patients who had preoperative creatinine levels of 2mg/dl and above were excluded from the study.

Statistical analysis of the data was done with SPSS for Windows with 95% confidence interval. Cathegoric data between groups was compared with Pearson Chi-Square and Fisher’s Exact test. Because continous data were not appropriate for normal distribution (Kolmogorov Smirnov P < 0.05) Mann Whitney U test for comparisons of two groups and Wilcoxon Signed Ranks statistical analysis were used for comparisons of preoperative and postoperative values. Values of P < 0.05 was accepted as statistically significant.

## RESULTS

Preoperative data of both groups did not differ statistically in terms of age, gender, presence of hypertension, diabetes mellitus, chronical renal insufficiency and smoking habbits (P > 0.05). However, there were more patients suffering from chronical obstructive pulmonary disease in group 1 (P < 0.05). In terms of preoperative bloodwork, creatinine, aspartate aminotransferase (AST) and alanine aminotransferase (ALT) levels were statistically similar (P > 0.05). However preoperative BUN level was statistically higher in Group 1 (P < 0.05) (Table-I). Also more patients were operated in emergency conditions and general anesthesia were preferred more in Group 1 than the other (P < 0.05). No statistical differences between the groups were seen in terms of postoperative hospital stay, pulmonary and other complications as well as mortality (P > 0.05) (Table-II). When the two groups were compared for blood parameters showing postoperative renal and other system functions, no statistical differences were detected (P > 0.05) (Table-III). However, when the groups were analysed for their preoperative and postoperative blood parameter changes, postoperative blood creatinine levels of the Group 1 were statistically decreased when compared to the preoperative levels but no statistically significant difference was found for Group 2. Postoperative AST levels were statistically higher in both groups when compared to the preoperative (P < 0.05). No such statistically significant difference was detected between preoperative and postoperative levels of BUN and ALT in neither groups (P > 0.05) (Table-IV).

**Table-I:** Preoperative patient data.

Preoperative Data	Group 1 n:95	Group 2 n:41	P
Age	73.5 ± 13.1	63.88 ± 23.9	0.058
Female Gender	63 (66.3)	29 (70.7)	0.613
Hypertension	65 (68.4)	22 (53.7)	0.100
Diabetes Mellitus	16 (16.8)	4 (9.8)	0.284
Chronical Obstructive Lung Disease	17 (17.9)	1 (2.4)	0.015
Chronical Renal Failure	9 (9.5)	2 (4.9)	0.504
Smoking Habbit	19 (20)	5 (12.2)	0.273
BUN	24.12 ± 11.51	19.54 ± 13.35	0.001
Blood Creatinine	1.26 ± 1.22	0.9 ± 0.53	0.054
AST	25.84 ± 15.14	35.59 ± 64.23	0.479
ALT	17.15 ± 9.87	28.04 ± 46.93	0.809

BUN: Blood Urea Nitrogen, AST: aspartate aminotransferase, ALT: alanine aminotransferase.

**Table-II:** Perioperative patient data.

Perioperative Clinical Data	Group 1 n:95	Group 2 n:41	P
Emergent Operations	69 (72.6%)	20 (48.8%)	0.007
General Anesthesia	38 (40%)	23 (59%)	0.045
Mortality	3 (3.2%)	2 (4.9%)	0.637
Postoperative Pulmonary Complication	11 (11.6%)	4 (9.8%)	1.000
Postoperative Other Complications*	17 (17.9%)	11 (26.8%)	0.237
Hospital Stay	12.56 ± 5.95	16.1 ± 12.12	0.447
Perioperative Blood Transfusion IU	1.2 ± 0.4	4.63 ± 1.62	<0.001

* These complications include liver dysfunction, infection, neurocognitive dysfunction, heart failure, deep vein thrombosis and pulmonary embolism.

**Table-III:** Postoperative blood parameters.

	Group 1	Group 2	P
Postop. BUN	22.91 ± 12.58	21.35 ± 11.86	0.519
Postop. Creatinine	1.17 ± 1.25	0.95 ± 0.82	0.221
Postop. AST	38.43 ± 70.43	41.05 ± 32.22	0.224
Postop. ALT	26.51 ± 75.9	23.02 ± 24.2	0.431

BUN: blood urea nitrogen, AST: aspartate aminotransferase, ALT: alanine aminotransferase.

**Table-IV:** Preoperative-postoperative differences in blood parameters.

	Group 1		Group 2	
	Mean ± SD	P	Mean ± SD	P
Preop. BUN	24.12 ± 11.51	0.320	19.54 ± 13.35	0.324
Postop. BUN	22.91 ± 12.58		21.35 ± 11.86	
Preop. Creatinine	1.26 ± 1.22	0.007	0.90 ± 0.53	0.595
Postop.Creatinine	1.17 ± 1.25		0.95 ± 0.82	
Preop. AST	25.84 ± 15.14	0.001	35.59 ± 64.23	0.005
Postop. AST	38.43 ± 70.43		41.05 ± 32.22	
Preop. ALT	17.15 ± 9.87	0.344	28.04 ± 46.93	0.498
Postop. ALT	26.51 ± 75.9		23.02 ± 24.2	

BUN: blood urea nitrogen, AST: aspartate aminotransferase, ALT: alanine aminotransferase.

## DISCUSSION

By the increase of surgical procedures in current health practices, perioperative blood transfusion incidence is also increasing. Besides financial burden many studies have also reported several medical complications associated with blood transfusions.^1^,^12^ Increase in postoperative complications like hemolytic and allergic reactions, transfusion-associated acute lung injury, transfusion-associated circulatory overload, graft-verse-host disease and infection as well as mortality due to blood transfusions in either orthopaedic or other fields of surgery are reported in numerous studies.^1^-^8^ Ponnusamy et al. recently published a review about effects of blood transfusion in orthopaedic surgery.^1^ Most common minor and major complications in this review were respectively allergic reactions (21%) and transfusion-associated acute lung injury (27%). Among these complications most common reasons of mortality were graft-verses-host disease (85-100%), transfusion-associated circulatory overload (2-15%) and transfusion-associated acute lung injury (5-10%). Many other studies also emphasize the increase in the risk of viral transmission and immunosuppression.^2^,^13^ Inconsistent with the literature, we did not find any statistical difference in rates of lung complications between the groups in terms of blood transfusion amounts in our study. Similarly, although other complications were also more common in highly transfused group, this difference was not statistically significant. However, at this point we have to state that comparatively common minor complications like allergic and hemolytic reactions were not recorded in our database.

Another complication of perioperative blood transfusion is deterioration in postoperative renal functions. This entity is not discussed much in orthopaedic literature but commonly referred in cardiovascular surgery reports.^9^-^11^,^14^ Kuduvalli et al. investigated the effects of blood transfusion on postoperative morbidity and mortality in coronary bypass surgery. They found 2.6% rate of postoperative acute renal failure in transfused patients compared to 0.2% rate in nontransfused which was statistically significant.^10^

Another study reports increased incidence of acute renal failure associated with transfusion of blood products in cardiovascular surgery. According to this study, acute renal failure incidence in transfused patient group was 8% compared to 1.8% in nontransfused group, and this difference was statistically significant.^9^ Godet et al. investigated the risk factors for postoperative acute renal failure in patients undergoing thorasic and thoracoabdominal aort surgery. They reported that mean transfusion amount of RBC was 11U in acute renal failure group compared to 7U in nonfailure, this difference was statistically significant.^11^

Results of our study, being one of the few studies investigating the effects of perioperative blood transfusion on kidney functions in orthopaedic operations, are inconsistent with cardiovascular surgery literature. Blood transfusion was reported to adversely affect postoperative renal functions in cardiovascular surgery, however similar effect was not proved in our study which focused on orhopaedic surgeries.

Consequently, there is no doubt that postoperative complication rates increase with perioperative blood transfusion. In contrast to other surgical disciplines we could not prove that blood transfusion in orthopaedic surgery had adverse effects on postoperative renal functions. However, we believe that larger prospective patient series including more parameters are needed to achieve more accurate results.

### Limitations of the study

It is a retrospective study, not all postoperative complications are recorded in the database, comparatively not a very large patient population and limited parameters indicating renal functions evaluated.
